# A Single RF Emitter-Based Indoor Navigation Method for Autonomous Service Robots

**DOI:** 10.3390/s18020585

**Published:** 2018-02-14

**Authors:** Tyrone Sherwin, Mikala Easte, Andrew Tzer-Yeu Chen, Kevin I-Kai Wang, Wenbin Dai

**Affiliations:** 1 Department of Electrical and Computer Engineering, The University of Auckland, Auckland 1023, New Zealand; tshe835@aucklanduni.ac.nz (T.S.); mikalaeaste@gmail.com (M.E.); andrew.chen@auckland.ac.nz (A.T.-Y.C.); 2 Department of Automation, Shanghai Jiao Tong University, Shanghai 200000, China

**Keywords:** autonomous service robots, industrial automation, indoor localisation, robot localisation, automated mapping

## Abstract

Location-aware services are one of the key elements of modern intelligent applications. Numerous real-world applications such as factory automation, indoor delivery, and even search and rescue scenarios require autonomous robots to have the ability to navigate in an unknown environment and reach mobile targets with minimal or no prior infrastructure deployment. This research investigates and proposes a novel approach of dynamic target localisation using a single RF emitter, which will be used as the basis of allowing autonomous robots to navigate towards and reach a target. Through the use of multiple directional antennae, Received Signal Strength (RSS) is compared to determine the most probable direction of the targeted emitter, which is combined with the distance estimates to improve the localisation performance. The accuracy of the position estimate is further improved using a particle filter to mitigate the fluctuating nature of real-time RSS data. Based on the direction information, a motion control algorithm is proposed, using Simultaneous Localisation and Mapping (SLAM) and A* path planning to enable navigation through unknown complex environments. A number of navigation scenarios were developed in the context of factory automation applications to demonstrate and evaluate the functionality and performance of the proposed system.

## 1. Introduction

As the prevalence of autonomous vehicles and robots (collectively referred to as robots in this paper) increases, the challenge of navigation in new and unknown environments (both indoor and outdoor) is a critical barrier to widespread adoption of these systems [1,2]. There are two key components that allow a robot to know where it should go—some form of mapping is required to know the positions of boundaries and obstacles to be avoided, and localisation is needed to allow the robot to know where it is relative to those movement constraints and how to best reach the target location. Simultaneous Localisation and Mapping (SLAM) techniques enable robots to achieve both of these tasks at the same time while operating within the environment [3]. Given a distant objective or goal location, using SLAM enables path planning and overall navigation of the system by dynamically improving the quality of the path over time to account for obstacles and other constraints [4,5]. The majority of existing indoor navigation systems [6,7] rely on vision-based approaches, which demand high computational power, costly equipment, and a stable environment without significant environmental changes over time. In cases where these obstacles cannot be visually seen using cameras or human vision due to a lack of line-of-sight, methods involving wireless signals need to be investigated instead, such as the use of the radio spectrum [8], sonar [9], or fixed points as virtual landmarks. However, this generally means that we need to deploy significant infrastructure to support these methods, such as multiple powered beacons with known positions. This is sometimes not possible in real-world applications, for instance, in factory or warehouse automation where raw materials or products may be placed at random locations and need to be delivered to other locations according to dynamic production schedules. In these ad-hoc cases, we still need to allow autonomous robots to perform some level of localisation in order to achieve tasks that otherwise may be challenging or time-consuming for humans, such as locating and fetching materials from a multi-storey warehouse or removing newly discovered hazardous waste from a construction site. Global Positioning Systems (GPS) may not always be available for localisation, particularly in an indoor context where the accuracy of GPS is much lower than in outdoor scenarios, or in a natural or human-caused disaster where satellite communications may be disrupted.

In this work, we propose a new approach of using a single emitter as a reference position in a global map, building on our previous work [10]. Such an emitter could be battery-powered and attached to a target object, giving an autonomous robot a target destination from its current location. In comparison to traditional RF-based approaches, we process the broadcasted messages to extract direction estimates relative to the robot’s current position in addition to the distance estimates. This is achieved primarily through a novel method that utilises multiple directional antennae to determine the direction of a single emitter beacon, relative to the robot, based on the Received Signal Strength (RSS). The direction estimates compensate for the inaccuracy of traditional RSS-based approaches that rely only on distance estimates. When combined with SLAM and our motion control algorithm, this enables a robot to manoeuvre towards that target without prior knowledge of the environment or any additional infrastructure deployment. Importantly, the emitter may not be in a fixed position, such as in a factory automation [11] or indoor delivery [12,13,14,15] context where targets may be mobile. We use a Pioneer 3-DX mobile robot [16] as a testbench for our method, with the goal of enabling the Pioneer robot to perform navigation by mapping a cluttered or unknown environment while localising itself relative to a single emitter of non-fixed position. Additionally, in order to enable navigation in a complex environment, there are situations where a robot needs to follow a path that initially moves away from the emitter to avoid large obstacles. This is achieved by integrating a heuristic A* path planning algorithm with our motion planning algorithm and SLAM to construct ideal paths to the target, while avoiding deadlock scenarios where the robot is trapped in a loop or stops navigation early before reaching the target destination.

The rest of the paper is organised as follows: Section 2 describes a target motivating scenario in the context of industrial automation in our work. Section 3 describes the proposed architecture of our navigation system and presents the Pioneer platform used for this research in more detail. Section 4 discusses works related to existing RF-based localisation, explains our unique approach that utilises a single emitter, and explores the use of filtering of the raw radio input data to improve target localisation. Section 5 covers our motion control algorithm that enables robots to manoeuvre without hitting obstacles. Section 6 covers the use of mapping and path planning for improved navigation in complex environments, and Section 7 presents the experiments and results along with full descriptions of the test cases and experimental settings. Section 8 concludes the paper and suggests future work for this research.

## 2. Motivating Scenario: Autonomous Factory Service Robot

**Figure 1:**
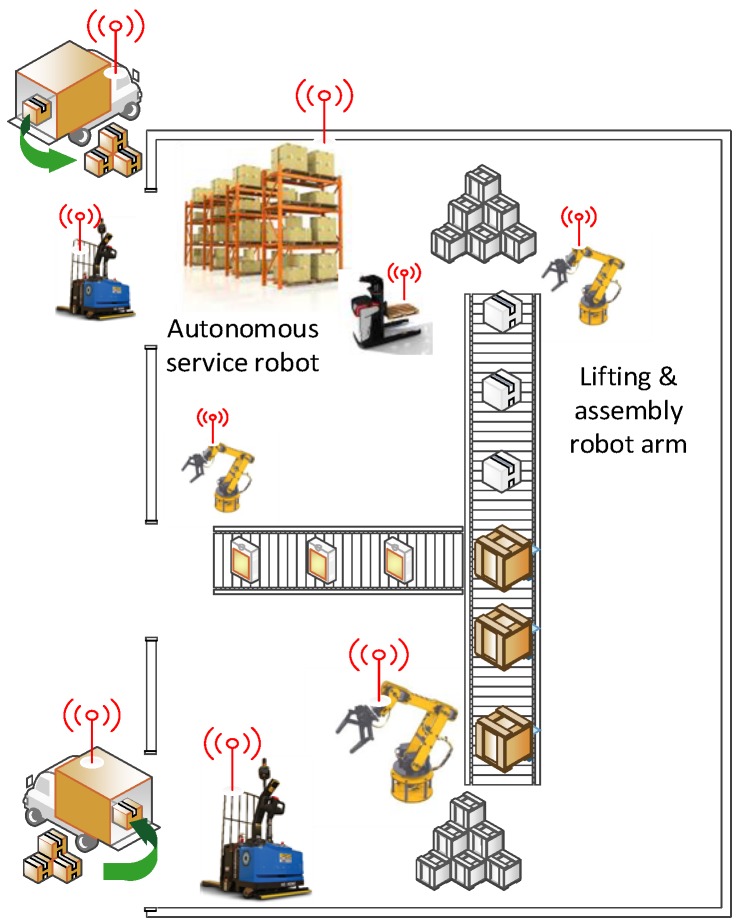
A diagram showing a factory automation application where raw materials and products need to be fetched from and delivered to different locations according to production schedules.

Our proposed emitter-based localisation and navigation approach can be applied to many real-world applications such as indoor delivery and industrial automation. In this research, we target a factory automation application as our motivating scenario. The main feature of this application is the requirement to navigate within a dynamically changing environment with minimal infrastructure deployment due to physical and/or financial reasons. Figure 1 shows a typical factory setting where items such as raw materials and final products need to be fetched from or delivered to different locations in a dynamic setting, depending on production schedules and logistics flow. In the vision of Industry 4.0 and the Industrial Internet of Things (IIoT), it is critical to be able to track the source materials and final products through technologies such as active RFID. In this context, it is not uncommon to assume that an RF emitter could be attached to a target object. Robots will need to have the ability to map and manoeuvre in a complex environment with multiple mobile and non-mobile obstacles. However, typically in a warehouse or production facility, the environment is structured in such a way that robots only need to travel in straight lines with 90° turns. It is also likely that there may be dead-ends in the environment, such as when rows of shelves are positioned against a wall. This means that there are cases where a robot could be in close physical proximity to a target object, but due to the presence of shelves or other obstacles, the robot cannot take a straight-line path and must move between different corridors or lanes in order to reach the target. This is beyond the ability of basic motion planning and manoeuvring, requiring long-term path planning. These requirements will be used to guide the design of our navigation system as well as the set of tests and experiments that are conducted for evaluating the system performance in Section 7.

## 3. Autonomous Robot System Design

Navigation for mobile platforms has been investigated extensively in the existing literature. In [17], a typical approach of combining computer vision, infrared range finders, and dynamic path planning is presented. In the context of industrial automation, [11] describes the use of 3D laser range finders, inertial measurement units (IMU), and a point cloud technique to dynamically map the environment and perform navigation. In [5], using laser range finders to detect artificial landmarks for localisation is investigated. Research such as [18] looks specifically at advancing long-term path planning in similar contexts.

**Figure 2:**
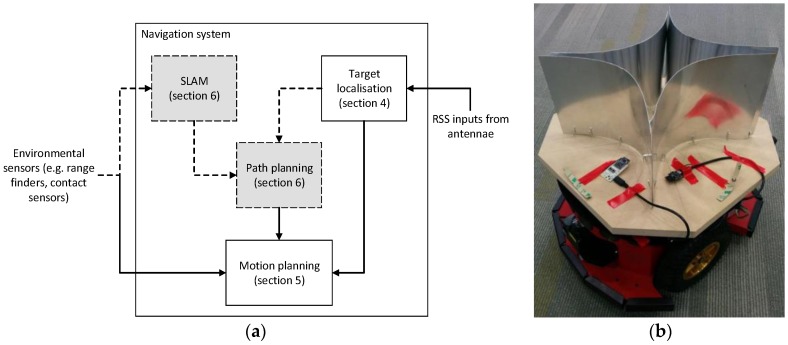
(**a**) System block diagram of the autonomous robot and (**b**) Prototype implementation on a Pioneer 3-DX research robot (© 2017 IEEE from [10]).

In order to achieve robust and accurate navigation towards a target object, we need hardware to support the goals of target localisation (identifying where the target is), robot localisation (identifying where the robot itself is), and environmental sensing (identifying where obstacles are). With this information, we are able to use software to generate a map of the environment, perform path planning, and determine how to control the robot’s actuators through motion planning in order to move the robot towards the target destination. The interaction between these different modules is shown in Figure 2a.

In this paper, we will present two versions of this system. In the preliminary (basic) navigation system, only the target localisation and motion planning modules are used, which are capable of navigating towards the target in simple scenarios but can become trapped in complex environments. In the full navigation system, all four modules are used so that the robot is capable of mapping the environment, constructing a long-term path plan to reach the final target, and manoeuvering to avoid obstacles in order to satisfy all requirements of the motivating scenario. The performance of these two navigation systems will be compared in Section 7.

In terms of sensing hardware, our system primarily uses a single wireless emitter (on the target) and four receivers (on the mobile robot) to perform the key task of target localisation, and some environmental sensors (i.e., a laser range finder and contact sensors) are used for basic obstacle avoidance. A simple method of obstacle detection is available through contact or touch sensors: when the robot comes into contact with an obstacle, it is detected and the system can react accordingly (generally by reversing and marking the obstacle in a map). However, this approach requires the robot to move slowly so that it has time to stop if it encounters an obstacle, and is still risky as a failed detection can lead to the robot colliding with an obstacle. Generally, range finding is more appropriate as it is a passive form of sensing that does not require physical interaction with the obstacle. By using some form of wireless signal, the distance between the robot and an obstacle can be detected by processing the signal in some way. There are a variety of methods that determine the distance to an obstacle [3,4]. The use of image processing with a camera allows for detailed information to be provided in a 3D space. Stereo vision can be used to find the depth of an area by comparing images of the environment from multiple points of view. In [19], a single camera is used in conjunction with odometry information from the mobile platform to compute depth information. However, vision-based methods are computationally expensive and may not be suited for embedded systems with limited computing resources available [4]. Alternative methods of range finding include sonar and laser range finding, which calculate the distance to an object based on the time of flight for an emitted signal to be reflected by the object and return to the emitter. Laser range finding provides higher resolution and accuracy than sonar for a greater initial cost of purchase. LIDAR systems can produce even higher resolution data [20], but these sensors generally attract a high cost, both computationally and financially [21]. In our system, we primarily use a laser range finder (Hokuyo, Osaka, Japan URG-04LX-UG01) for obstacle detection, with contact/touch sensors as a failsafe in the event that the laser range finder fails.

Since we assume that GPS is unavailable in the targeted indoor factory automation context, there is no global object tracking system available to keep track of the position of the robot, so we must use another form of robot localisation [2]. As the Pioneer robot used in our research has wheel-based locomotion, we can use wheel encoders to provide odometry data for this purpose. Finally, we require some form of actuation so that we can move the wheels in a way that allows the robot to make progress towards the target destination. This is provided by two electric motors inside the robot, driving the wheels on each side.

This system has been implemented on a Pioneer 3-DX research robot, which is shown in Figure 2b with four radio receivers and parabolic reflectors. The robot has an HP Mini 110-3627TU N570 netbook with a 1.66 GHz processor and 1 GB of RAM on board with Ubuntu 14.04.1 LTS, which runs the software for this robot. Robot Operating System (ROS) [22] was used to interface with the robot itself, which runs as a series of nodes that communicate using messages. The robot itself and each sensor were abstracted into ROS nodes, and each of the four software modules exists as an individual node to allow for greater modularity and reduced coupling.

## 4. RF-Based Target Localisation

### 4.1. Related Works

In order to effectively determine the location of the RF emitter as a navigation goal, a method of RF ranging was chosen. Traditionally, RF-based ranging includes Received Signal Strength (RSS), Time Of Arrival (TOA), Time Difference Of Arrival (TDOA), Angle of Arrival (AOA) and Phase Difference Of Arrival (PDOA) [23,24]. Each of these has its own advantages and disadvantages. RSS works by using the amount of power lost over a distance by an RF signal. It has the advantage of giving an estimate in almost all cases and being easy to implement, but it is known to fluctuate and is heavily affected by multipath effects and interference [25,26]. Time of Arrival is implemented by measuring the time taken for a signal to propagate between two nodes. This is typically more accurate than RSS when the two nodes have a clear line-of-sight, but accuracy decreases significantly without line of sight [27]. TDOA compares the time of arrival between a node with a known location and the node to be localised. This is accurate if well calibrated, but requires a sufficiently large distance between the two nodes for the difference in arrival time to be perceptible for fast moving signals [9]. The phase difference of arrival measures the phase at the node to be ranged and a known node, and uses the difference between these two to calculate the distance [23]. In this work, we use AOA, which is measured by using one of these methods of ranging over an array of antennae, and then apply basic trigonometry to work out the angle of origin [24]. The number of antennae needed can be reduced through the use of directional antennae. When these antennae are oriented in different directions, the difference in RSS between them can then be used to find the AOA. Using directional antennae is only needed when using RSS to determine range, as the other ranging methods are not affected by the type of antenna [26]. Summaries of various localisation technologies with their measurement techniques and accuracy are available in [23,28].

There are a number of existing studies that use RF media for localisation. One example provided in [29] uses the RSS of Wi-Fi signals to track a moving object by detecting changes in the signal strength. In [30], three systems that use Radio Frequency Identification (RFID) tags for 3D real-time localisation are presented. In [8], a pseudo-Doppler method is used to locate RF emitters using a sensor platform that detects the line of bearing (LOB) to the emitter. Rapid switching between multiple antennae is used to find the phase difference which is then converted into a directional angle. By using multiple autonomous platforms, they can communicate and send information to each other in order to optimise their paths and determine the location of the emitter. This project aims to achieve a similar goal; however, multiple robots can be very costly and unsuitable for some applications. Therefore, we aim to achieve the goal of determining the angle to the emitter using a single robot by measuring the RSS in multiple directions and using the strongest, and therefore most likely, signal as the direction of origin.

### 4.2. Design Concept and Methodology

A common issue with RSS-based signal analysis is the potential for multipath interference [25,26], i.e., interference caused by reflections of the transmitted signal resulting in ghosting and fading of the signal. Reflections additively interfere with the underlying signal, which can be constructive or destructive depending on the phase of the signals, artificially increasing or decreasing the signal strength away from the true value. This makes it challenging to determine the true distance to an emitter under environments where there is no line-of-sight, or where there are many obstacles or reflective surfaces that cause reflections, as this affects the signal strength.

In our approach, we focus on estimating the direction of the emitter relative to the receiver to compensate for inaccurate distance estimations from an RSS-based approach. In [31], directional antennae are used to determine the angle of arrival based on the RSS of a signal. We form a rudimentary directional antenna by using a parabolic reflector dish with an omnidirectional antenna. The reflector dish concentrates the signal and focuses the gain in a single direction towards the receiver. Parabolic gain, *G*, is proportional to the ratio of the area, *A*, of the dish over the square of the wavelength, *λ*, following the Equation:

(1) $$G=[4\pi A/(\lambda^{2})]e_{A}$$

where $e_{A}$ is a dimensionless, catch-all parameter between 0 and 1 called the aperture efficiency. This parameter accounts for any and all imperfections in the aperture including the curvature, spill-over from an incomplete dish, and blockage by the antenna itself. Typical values for $e_{A}$ are between 0.55 and 0.70 [32]. From Equation (1) it can be seen that decreasing the wavelength by a factor of 2 requires an increase of 4 in area to achieve the same directional gain. The RF sensor nodes used in this project have a radio frequency of 915 MHz and a wavelength of 32.76 cm.

In this research, the longer transmission distances achieved from a higher gain are less important than resultant directionality from the beam width of the radiation pattern. In particular, the half-power beam width (HPBW, *θ*), i.e., the angle between the points of the antenna radiation pattern at which power drops to one half (−3 dB) of its maximum value, is very important and is given for a circular dish with diameter *d* by

(2) θ=kλ/d

where the *k* factor for a typical parabolic antenna is approximately 70, varying slightly depending on the shape of the reflector and the feed pattern.

The beam width property of parabolic antennae leads to different readings in multiple directions (i.e., antennae have better directionality), which allows us to better determine the angle of arrival of a signal by comparing these values [31]. Since the gain and beam width are inversely related, we can increase the gain to decrease the beam width and improve overall system performance.

Instead of using a single high-cost, high-resolution sensor (i.e., an RF antennae offering RSS data stream in our case), we use a sensor array of multiple cheaper sensors to emulate the same behaviour. Following the work of Smith et al. [33], we use multiple, overlapping sensors as an effective means of obtaining accurate results. By using multiple antennae, this also means that we need multiple parabolic reflectors. This creates a limitation in that we are constrained by the amount of space available on the prototyping robot platform since each reflector requires a large amount of space, therefore limiting the magnitude of directional gain that can be achieved.

By using paired antennae, we are able to perform simultaneous readings with 180° offsets, providing two opposing points of reference. This is in contrast to using a single sensor, which could also provide multiple points of reference, but with slight timing differences limited by mechanical constraints. Our method has the additional benefit of improving resilience to noise and sudden changes in the environment that can cause erroneous readings to occur when there are time offsets between readings. Using paired antennae in this way avoids the need to determine the radiation pattern of the antenna, as we can use the instantaneous values for mapping the RSS into a probabilistic direction. To provide sufficient resolution and directionality in a two-dimensional planar coordinate space, a minimum of two pairs should be used at a 90° offset between each pair, meaning that there should be a total of four antennae that are perpendicular to each other.

**Figure 3:**
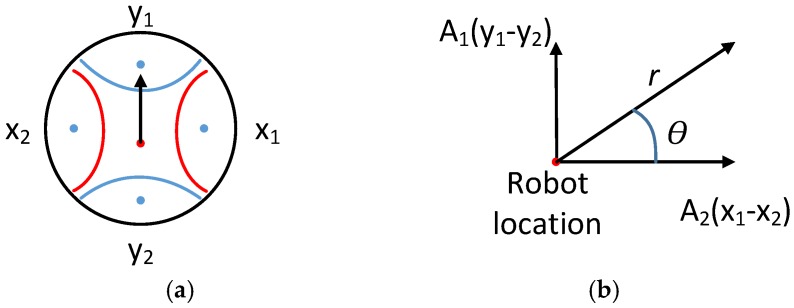
(**a**) Top view illustration of the physical arrangement of two pairs of antennae with their reflectors aligned along *x* and *y* axes, and (**b**) calculation of the estimated relative direction *θ* and distance *r* based on differential RSS.

**Figure 4:**
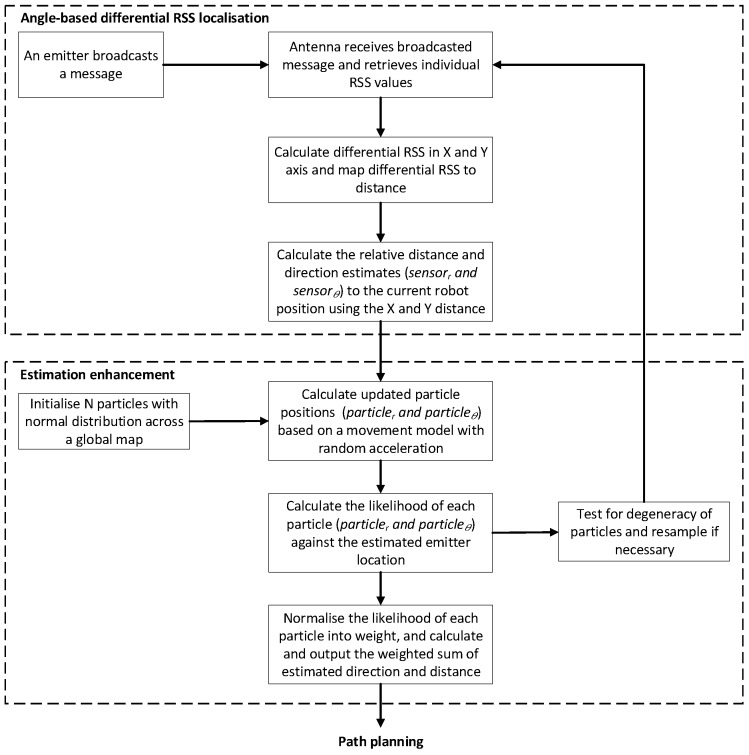
Angle-based differential RSS localisation algorithm.

Based on this design concept, the proposed angle-based differential RSS localisation approach is introduced here. Figure 3a illustrates how the two pairs of antennae with their reflectors are arranged in the *x* and *y* axes for two dimensional localisation. Referring to Figure 3a, *y*_1_, *y*_2_, *x*_1_ and *x*_2_ represent the individual RSS values received from the emitter, and the black arrow indicates the current heading direction of the robot. After calculating the differential RSS value for each axis (i.e., *x* and *y* axis), we follow the traditional approach of mapping RSS values to distance. Parameters A1 and A2 are introduced after calibrating the differential RSS with distance mapping. The relative angle, *θ*, and distance, *r*, between the emitter and the robot are calculated using Equations (3) and (4), and illustrated in Figure 3b. Referring to Figure 4, the angle-based differential RSS localisation is designed to provide reasonably accurate direction and distance estimates. The estimates are further enhanced using a particle filter in order to reduce the inherent fluctuations of RSS-based estimation. The differential localisation method is validated in Section 4.3, and the estimation enhancement is explained in Section 4.4.

(3) θ=arctan((x1−x2)(y1−y2))

(4) $$r=\surd \left({\left(x_{1}-x_{2}\right)}^{2}+{\left(y_{1}-y_{2}\right)}^{2}\right)$$

In our current prototype, we used off-the-shelf components to create a simple, low-cost directional antenna using a 915 MHz omnidirectional antenna and a custom-made cylindrical parabolic reflector dish. Using an emitter at a fixed location, we can verify the directionality of the antenna by running an experiment that measures the RSS and compares it to known direction values. We used an STM32 ultra-low-power ARM-based processor with a low-power Atmel AT86RF212B transceiver to process the signals from the antenna and provide the necessary RSS values [34].

### 4.3. Angle-Based Differential RSS Localisation Validation

**Figure 5:**
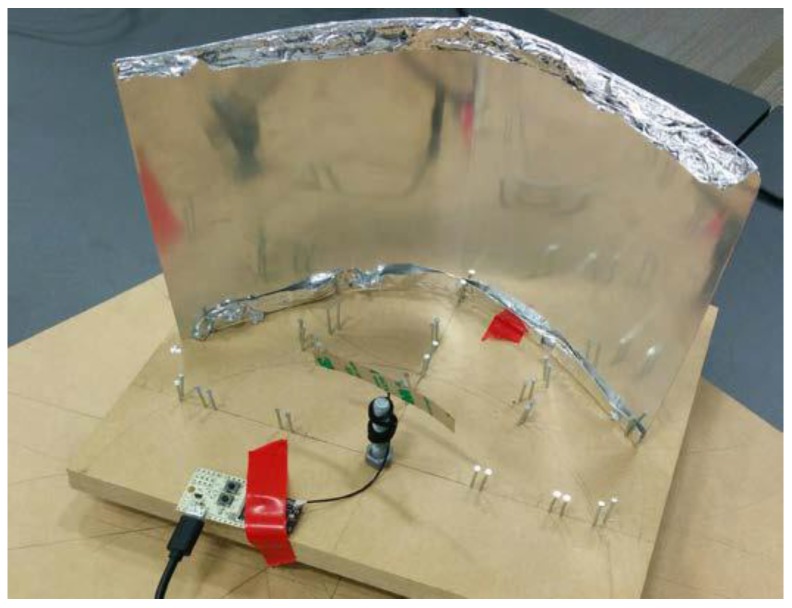
Test prototype for testing the gain of a single receiver node which is mounted on a rotational platform at a fixed position relative to the transmitter, but allowing varying orientation.

A test prototype, shown in Figure 5, was constructed for the experiment. The prototype was able to be rotated through a full 360° of motion, with points marked at 30° increments for testing RSS in different directions. Mounting points were provided for the reflector dish and receiver such that the receiver was both in the focal point of the dish and the centre of rotation. The parabolic reflector dish was constructed from an aluminium sheet of 0.5 mm width, and aluminium foil was added to some tests to provide shielding on the reverse side. Tests were carried out for a reflector of length 300 mm and height 200 mm, shaped to radii of 80 mm, 130 mm, and 150 mm. Additional tests were also conducted without a reflector in order to compare the improvement in directionality against a control.

**Figure 6:**
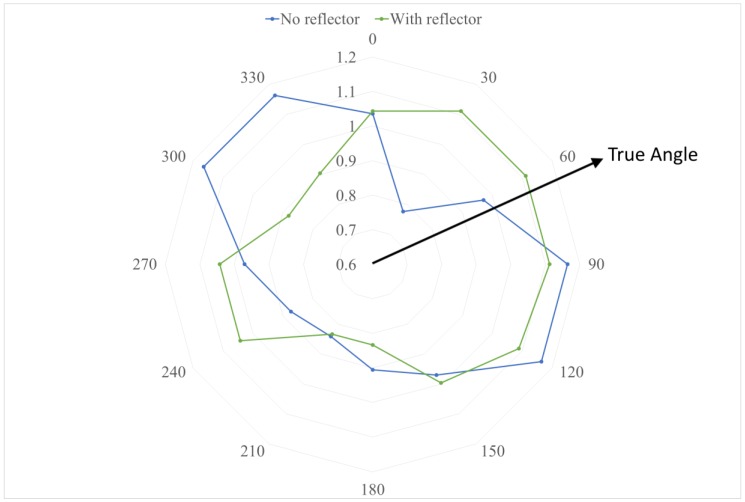
Comparative analysis of the Received Signal Strength (RSS) for an antenna with and without a reflector. The RSS (normalised as a ratio of the mean value) shows improved directionality, with the strongest RSS in the direction of the true angle when using a parabolic reflector.

The transmitter (i.e., the emitter) was positioned in a fixed location relative to the receiver. This allowed the test prototype to be rotated while measurements were taken and values recorded. A number of tests were performed, varying the reflector radius as well as the positions of both receiver and transmitter. For each test, the RSS results were tabulated and graphed against the angle. In addition to the monopole antenna shown in Figure 6, tests were also carried out with a Splatch ISM antenna to demonstrate the feasibility and effectiveness of this method.

Our test results indicate that adding a parabolic reflector improved the directionality of the antenna, as can be seen in Figure 6, which illustrates an example case. As expected from the supporting theory, the reflector with the largest aperture size provides better performance and was able to achieve an error of ±15 degrees. However, this error is also due to the fact that the step size of the angle increments was selected to be 30 degrees, so this is essentially the minimum precision.

Once these tests were completed, a second node was added for the purpose of testing two paired receivers in order to measure the difference in signal strength. Testing of the paired receivers included trials with no reflectors and tests where each antenna had a reflector of 130 mm radius similar to the above. By using paired receivers, this test was more similar to the intended use of the antennae. When reflectors were used, the absolute differences at opposing orientations (i.e., the difference between the two receivers aligned approximately with the transmitter) indicated a significant difference in power received. This difference could then be used to isolate the direction of origin of a signal, and thus the direction of the target emitter.

Using the RSS signal, we are also able to derive an approximate measure of distance to the target emitter. However, this is very unreliable due to the presence of noise and multipath effects, so we only use this for the purposes of path planning for setting a target destination. In the case where no path planning is used, the robot will simply navigate in the target direction, until the distance is sufficiently and reliably small to give us confidence that the robot has arrived at the destination.

### 4.4. Enhancing Angle and Distance Estimation

The main shortcoming of using a Received Signal Strength (RSS)-based approach is that RSS is very susceptible to multipath effects that can cause highly fluctuating and noisy readings. The situation is further exacerbated when the source and/or emitter are moving. Therefore, we cannot use the raw instantaneous RSS values and should filter out as much noise as possible in order to maintain a reasonable level of accuracy while achieving real-time processing. Since we have a mobile platform, we can take advantage of the historical values to process data points collected at different positions, minimising the effect of localised noise and other variations in RSS. By combining the information collected at different positions, the accuracy can be improved by attempting to isolate the underlying true signal which should be consistent at different positions. This also reduces the likelihood of local optima causing the system to move in the wrong direction.

There are multiple possible methods of filtering the signal for estimating the true position of the emitter. The most basic method would be simple averaging; however, this does not take the movement of the robot into account, so it would be less accurate [35]. Due to the nature of RSS, uncertainty will be inherently high, so we have used a Bayesian approach. One of the most common Bayesian approaches for localisation is the Kalman filter and its variations [36]. A Kalman filter makes predictions about a number of possible future states based on the current state, then updates the probabilities of these predictions based on the sensor readings at that future state [37]. An alternative to a Kalman filter is a particle filter [36]. When used in localisation, a particle filter simulates a number of possible points in the environment, each with a position and velocity. Each particle is weighted based on the probability that it is the true point. Low probability particles are removed, and the remaining particles should converge on the true point. Both the Kalman filter and particle filter determine the probability of each possible state, given the sensor readings based on Bayes’ theorem [38]:

(5) P(A|B)=P(B|A)P(A)P(B)

Bayes’ theorem states that the probability of *A*, given *B*, is equal to the probability of *B*, given *A*, multiplied by the probability of *A*, normalised by the probability of *B*. In localisation, this is typically used as the probability of being at position *A*, given sensor data *B*. For linear Gaussian applications, the Kalman Filter produces an exact approximation of the true point. However, in non-Gaussian applications the Kalman filter is much less accurate [36], so for this application, the particle filter has been chosen. The particle filter steps are illustrated as the estimation enhancement part of the algorithm in Figure 4.

**Figure 7:**
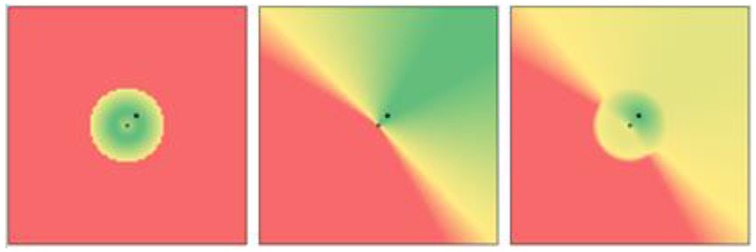
Visualisation of the particle weight functions for a ground truth location of (4, 4), meaning a displacement of 4 m in the x and y directions from the receiver, indicated by the dark square. The receiver is in the centre of each image at (0, 0). The weight function based only on distance is shown in the left image, and the weight function based only on the angle (direction) is shown in the middle image. The final combined weight function is shown in the right image. Red indicates a low likelihood, and green indicates a high likelihood of the emitter position matching the particle location.

At initialisation, *N* particles are generated based on the starting location of the robot in the global frame (i.e., the coordinate system offered by the path planning module). The particle positions are updated in each iteration according to a movement model with random acceleration [35] when there are new RSS measurements. The weight (i.e., the likelihood) of each resultant particle is calculated based on both distance and direction. The distance and direction of the emitter based on the instantaneous RSS readings are calculated using Equations (3) and (4), and the difference between these values and the position of the particle determines the weight elements using Equations (6) and (7), where *r* is the distance from the robot and *θ* is the angle offset from the robot’s current heading.

(6) $$w_{r}=1.0-t_{r}\times \left(\left|particle_{r}-sensor_{r}\right|-c_{r}\right)$$

(7) $$w_{\theta }=1.0-t_{\theta }\times \left(\left|particle_{\theta }-sensor_{\theta }\right|-c_{\theta }\right)$$

where *sensor_r_* and *sensor_θ_* are estimates of the current relative distance (in meters) and relative angle (in degrees) of the emitter based on the instantaneous RSS measurements, *t_r_* and *t_θ_* are tolerance levels (set to 0.1 and 0.01 respectively for our prototype), and *c_r_* and *c_θ_* are constant offsets (set to 1.0 and 15.0 respectively for our prototype). The tolerance level represents error in these estimates, adjusted according to our expected accuracy of the measurements. In the proposed algorithm, we focused on using direction estimation to compensate for erroneous distance estimation, and therefore *t_r_* is 10 times higher than the value of *t_θ_*, i.e., distance estimation has a higher error tolerance. The constant offsets represent a minimum threshold that must be crossed before the measurements start to affect the weights in order to avoid fluctuations caused by noise. In our case, the distance difference needs to be larger than 1 m (i.e., *c_r_* = 1) and the angle difference needs to be larger than 15 degrees (i.e., *c_θ_* = 15) to start influencing *w_r_* and *w_θ_* respectively. These weight elements are then clamped so that any value larger than 1 is set to 1, and any value smaller than 0.1 is set to 0.1. The particle weight is calculated by determining the square root of the product of the two weight elements as shown in Equation (8). The square root was taken to ensure the scale of the weight distribution is between 0.1 and 1, avoiding extremely small weights (i.e., less than 0.1) that may cause undesirably fast convergence towards suboptimal estimates. An example of how combining the distance and direction weight distributions leads to more accurate overall estimates is shown in Figure 7.

(8) $$weight=\surd (w_{r}\times w_{\theta })$$

The weights of all particles are then normalised into probabilities so that the sum of all particle weights is equal to one, as shown in Equation (9). The final estimated direction and distance are calculated according to Equations (10) and (11) by calculating the weighted estimated direction and distance from all particles to reduce the impact of significant fluctuations caused by raw RSS measurements. In order to avoid a model with a very small number of high probability particles, a resampling step occurs [36]. This is triggered when the inverse of the sum of the squares of the weights is less than half the total number of particles, as indicated in Equation (12):

(9) Pi=weighti∑i=0Nweighti

(10) $$Estimated_{r}=\sum_{i=0}^{N}particle_{r}^{i}\times P_{i}$$

(11) $$Estimated_{\theta }=\sum_{i=0}^{N}particle_{\theta }^{i}\times P_{i}$$

(12) $$\frac{1}{\sum_{i=0}^{N}P_{i}^{2}}<\frac{N}{2}$$

After the resampling step, the number of particles remains the same, *N*. However, the new particles are sampled from the existing particles, where the probability for each existing particle to be sampled is the weight of that particle. This has the effect of increasing the number of higher weight particles and decreasing the number of lower weight particles, which leads to convergence of the filter. The number of particles to use is based on a trade-off between accuracy and computational load, with O(*N*) linear complexity for *N* particles. An increase in particles reduces error and provides better spread. Better spread reduces the chance of missing the true point and converging on an incorrect local maximum. In our case, we used 1000 particles. The target point provided by the particle filter and used in path planning is a weighted sum of all the particles, as shown in Equations (10) and (11).

**Figure 8:**
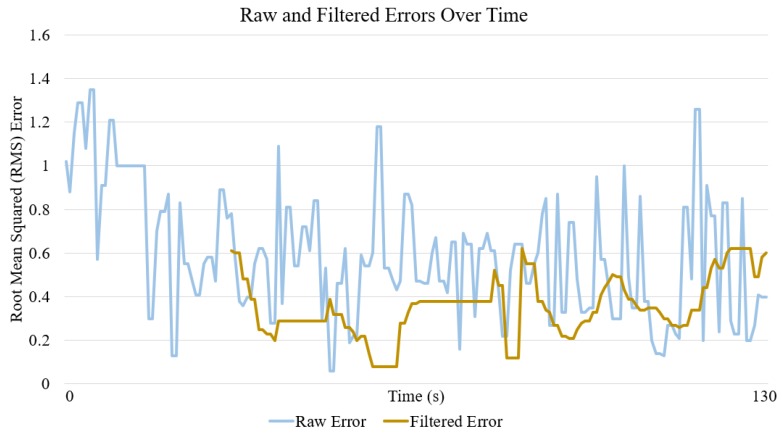
Raw and filtered errors over the course of a benchmark test.

When comparing the error in the instantaneous position value calculated directly from the antennae, and the error in the filtered position estimate, we found that filtering reduced the average error by up to 50%. An example is illustrated in Figure 8, where the root mean squared error of the position has been plotted over the course of a benchmark test.

## 5. Motion Planning

The readings from the environmental sensors (i.e., the laser range finder and contact sensors) provide information about obstacles in the local context, which can also be placed onto a global map. In the initial case where there is no map, these sensors play a major role in ensuring safe navigation by avoiding obstacles while the robot drives directly towards the estimated target location and the map is being formed.

We mounted the directional antennae and parabolic reflectors on top of the Pioneer as shown in Figure 2b. This allowed the target localisation module to use the RSS readings while the Pioneer moves in order to determine an approximate direction of travel. By comparing the relative strengths along the two axes, an approximation for the distances in each axis can be found. Through simple trigonometry, these distances can then be combined to find the distance and direction of the target emitter from the current position. However, the derived distance is generally very inaccurate, as it is strongly influenced by noise and multipath effects, so we primarily focus on using the direction for motion planning purposes. Collectively, the location information and environmental sensor inputs are used in our motion planning algorithm to control the local movements and gradually move towards the target location.

**Figure 9:**
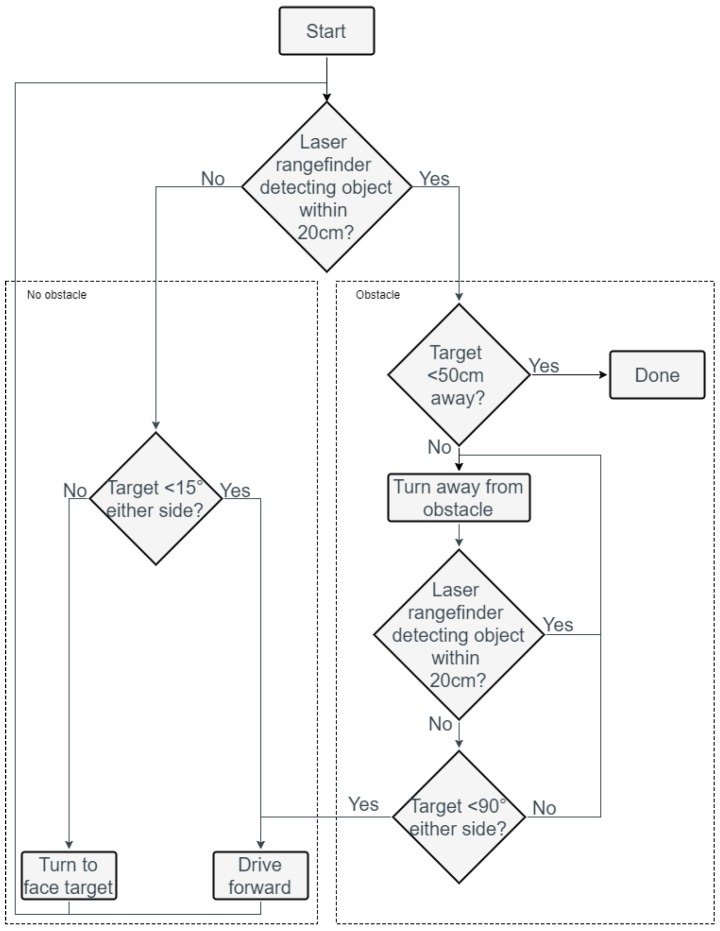
A simplified overview of the preliminary system control flow chart.

In the initial case when the robot is first turned on, we follow a simple heuristic for safe navigation. If there is no obstacle within 20 cm of the laser range finder, and the angle to the target is within 15° to either side of its current heading direction, then the robot will drive forward. Otherwise, the robot will gradually turn towards the target while driving forward. If there is an obstacle within a 20 cm range, then the system enters its obstacle avoidance mode. In this mode, the robot will continue to turn until there are no obstacles detected within a 20 cm range of the rangefinder. When the obstacle is no longer in front of the robot, the robot will continue to move forward if the target is in the forward direction of the robot (i.e., target is located less than 90° either side relative to the current robot heading direction). When the sum of the front and back RSS values is above a certain threshold that indicates a distance to the target that is less than 50 cm, we consider the robot to have reached the target destination (since the robot has a diameter of 40 cm), causing the robot to stop. An overview of this motion control algorithm is given in Figure 9. In addition, if any of the front contact sensors detect an object then the robot will reverse immediately to avoid the object. Similarly, if any of the rear contact sensors detect an object then the robot will drive forwards.

With this preliminary implementation, we verified that the wireless emitter could be used as a point of reference, and that our RSS-based algorithm with angle approximation provided an accurate target direction. In scenarios where there is a direct path to the emitter, this method is sufficient for reaching the goal. In some scenarios where the direct path is partially blocked, the system is able to use the variance in the received signal and simple local obstacle avoidance to reach the goal destination. However, due to the simplistic nature of the motion planning, and without any memory of the position of obstacles this system, the system is unable to recognise dead ends and is susceptible to being trapped in corners. This is quite common in the context of factory automation, where the strength of the emitter could be very strong indicating the target is near, but it is not actually directly reachable due to obstacles such as conveyor lines and shelves. Therefore, introducing mapping and long-term path planning into the system is required in order to find viable paths and avoid deadlock.

## 6. Mapping and Path Planning

### 6.1. Simultaneous Localisation and Mapping (SLAM)

In order to enable long-term decisions and allow for smarter path planning with obstacles, we use a SLAM module to map the environment by processing the odometry data and laser range finding provided by the Pioneer robot. By comparing the distance to detected objects at multiple locations using the odometry information to approximate the distance between scan locations, obstacles can be found and tracked in the environment.

Simultaneous Localisation and Mapping (SLAM) has been an active research area for many years, with significant progress made towards universally applicable solutions [3]. Since our focus is on ascertaining the direction of the goal destination based on a single emitter and not on localisation or mapping of the robot itself, we used a third party tool, GMapping, for the SLAM module. More information about the approach used in GMapping can be found in [39].

**Figure 10:**
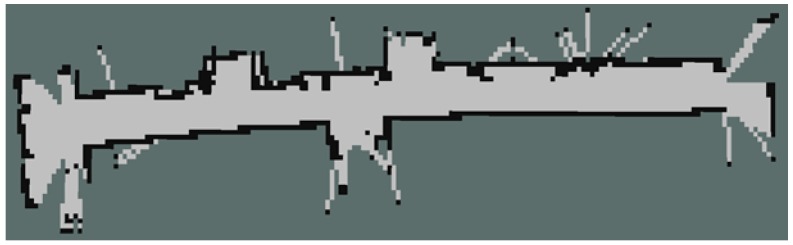
An example occupancy grid output visualisation.

Gmapping produces an array that discretises the world into grid cells. Each grid cell is then filled with 100, 0, or −1 representing occupied, empty, and unknown respectively. These are populated within Gmapping based on information gained from sensor data. In our implementation, each of these grid cells represents 400 cm^2^ of real-world space (20 cm on each side). A visualisation of the occupancy grid is shown below in Figure 10.

**Figure 11:**
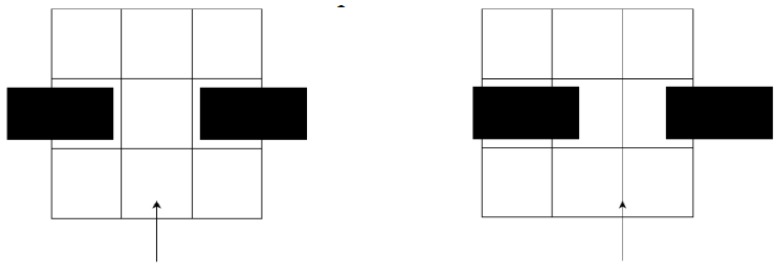
Example of how a valid path can be interpreted as both valid and invalid.

The grid cell size is selected based upon a trade-off of having more information to navigate with against a higher computational load. Since each grid cell can only be empty or occupied, if the grid size is too large, a valid path may not be recognised, depending on the alignment of the grid. An example of this is shown in Figure 11, where the left image shows a situation where the robot, represented by the arrow, will determine that there is a valid path between the two obstacles. The right image, however, will register all of the grid cells in the row above the robot as occupied, and the robot will not believe there is a valid path. Therefore, it was necessary for the size of each cell to be slightly smaller than what the robot can drive through. 

This can be resolved by decreasing the size of the grid cell so that it was smaller than the width of the robot (40 cm in diameter). This reduces the number of false negatives where the robot does not recognise a valid path, but introduces false positives where the robot will recognise empty space that it cannot actually fit through. This is accounted for separately by the path planning module with local obstacle avoidance.

### 6.2. Path Planning

In our current system, the navigation is separated into two components representing the macro and micro level decisions: path planning and motion planning. The path planning component combines the local area information from the environment map and the target localisation information from the RSS data streams together. The path planning module uses this information to determine the path to be taken by the robot in order to reach the desired goal and avoid being trapped in dead ends. For macro-level decisions, the map of the local environment created by the SLAM module is used, with the current estimated target position placed on this map. The path planning module will then attempt to find a path from the current position to the target position.

There are many different methods of path planning. The shortest path algorithms most commonly used include breadth-first search, Dijkstra’s algorithm, and A* [40]. Breadth-first search involves branching to all of the neighbouring cells until the goal is found [41]. Dijkstra’s algorithm behaves like breadth first, but also calculates the cost of traversal to each point, selecting a path that has both the shortest distance and the lowest cost [40]. This is beneficial when not all grid cells have the same cost, such as paths that need additional manoeuvring procedures or avoiding obstacles. The A* algorithm also uses the cost of traversal of each cell in the same manner as Dijkstra’s, but additionally determines which neighbouring cell to explore first depending on a heuristic. This heuristic is typically an estimate of the distance to the goal. This allows the algorithm to be directed toward the goal and is therefore more efficient. As breadth-first search does not allow cost to be based on more than just distance, this is not well suited to this application. Since A* with its heuristic is more efficient than Dijkstra’s algorithm, we selected A* for the purpose of this research to find the shortest path. For simplicity, the movement was locked to the grid cells of the SLAM occupancy grid. This means that the robot only needs to move in one of the four cardinal directions relative to the starting point, rotating in 90° increments and driving straight forward or back as needed.

The A* pathfinding algorithm uses a best first style search, prioritising exploration of paths which give the lowest total estimated cost. When calculating the total estimated cost, the algorithm sums the current cost of a path with the estimated remaining cost. This remaining cost uses heuristics for guiding the search to reduce the time taken to find the optimal path. The heuristic uses the Manhattan distance between the current position of the path and the target position, giving the total difference in x and y positions. This provides a reasonable estimate of the remaining cost when the cost of a path is determined by the number of cells traversed and ensures the optimal known path is found.

**Figure 12:**
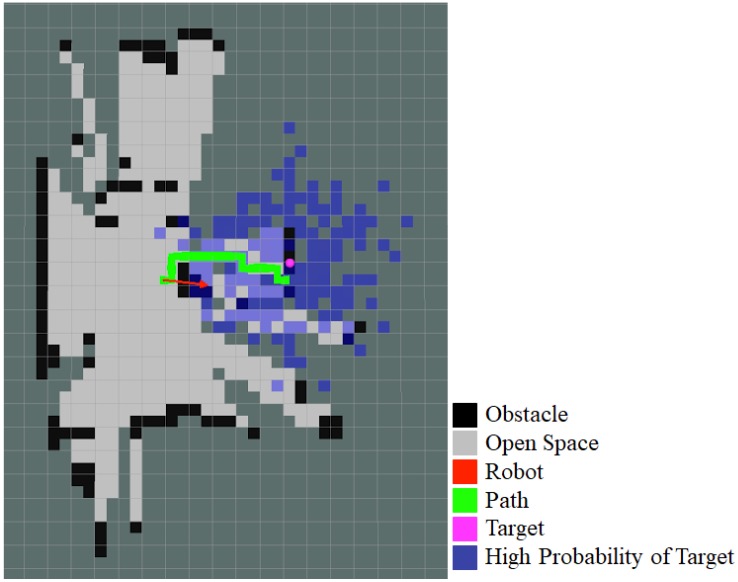
Visualisation of the map, particle filter, and path.

The implemented A* algorithm takes into account a number of factors in addition to the number of cells when determining the cost of a path. Each map cell has a cost of 100, 1, or 2, depending on if the cell is occupied, unoccupied, or unknown, respectively. This incentivises taking a path with known occupancy over a path with unknown cells. As each grid cell is smaller than the robot, there is a possibility that there may be a path that is considered to be viable that the robot cannot actually fit through. In order to mitigate this, the map should assign a higher cost to cells with multiple obstacles near them. This is calculated by summing the cell in question, along with its four adjacent cells. This encourages the robot to take paths that are further away from obstacles, which may lead to slightly less direct paths but decreases the likelihood of taking an invalid path and getting stuck. Additionally, if the space between obstacles is less than three grid cells, then the values in these cells are essentially high enough to prevent a path from being planned through this space, ensuring that the robot avoids those obstacles. Lastly, due to the simplified movement requiring alignment to cardinal directions, turning to change the direction of motion requires coming to a complete stop and turning until the correct direction is reached. This manoeuvre time is included as a type of cost in the path by using the current orientation as a proxy, so that the robot will also prefer paths that have fewer changes in orientation, even if this means that the actual path is longer. Figure 12 shows a visualisation of these different components. This includes the map, in grey and black, the particles from the particle filter in blue, and the weighted sum of these particles in pink. The robot is represented by the red arrow, with the arrow pointing in the target direction. The planned path from the red arrow to the pink target is visible in green; it can be seen that there is an obstacle in front of the robot and that the path diverts around it.

During exploration of the unknown areas, the SLAM module maps these areas and assigns values based on the occupancy of the cells. Since the path planning is executed periodically, the new information is taken into account and the path is updated over time in a way that supports continued exploration until a valid path is found to the target destination. The target destination is defined by both the direction and distance to the target emitter. However, we mostly focus on the direction, as the distance measured is often inaccurate, and so we rely on iterative improvements to the planned path by running the module periodically to update the path as the robot progresses towards the goal.

With SLAM and path planning, the motion planning module described in Section 5 was then expanded upon to allow for execution of the path provided by the path planning module, as shown in Figure 2a. By providing the odometry data to the motion planning module, it is possible to determine the current location on the map, and thus the current point in the path. By comparing the path’s current position and next position, the robot can calculate the desired bearing of motion, turning as necessary to reach it. Once facing the right direction, the system will continue to move forward until this direction changes.

In the event that the robot finds itself off of the path provided, it will fall back to the preliminary system (i.e., using only target localisation and motion planning modules) for finding the target. This can sometimes occur when the map output from the SLAM module experiences a discontinuity, causing a discrete jump in the position of the robot relative to the map. This generally only occurs as a result of drift in odometry when the robot rotates, as the drift for regular motion is much lower and does not require the same degree of compensation.

## 7. Navigation Testing

### 7.1. Testing Scenarios

**Figure 13:**
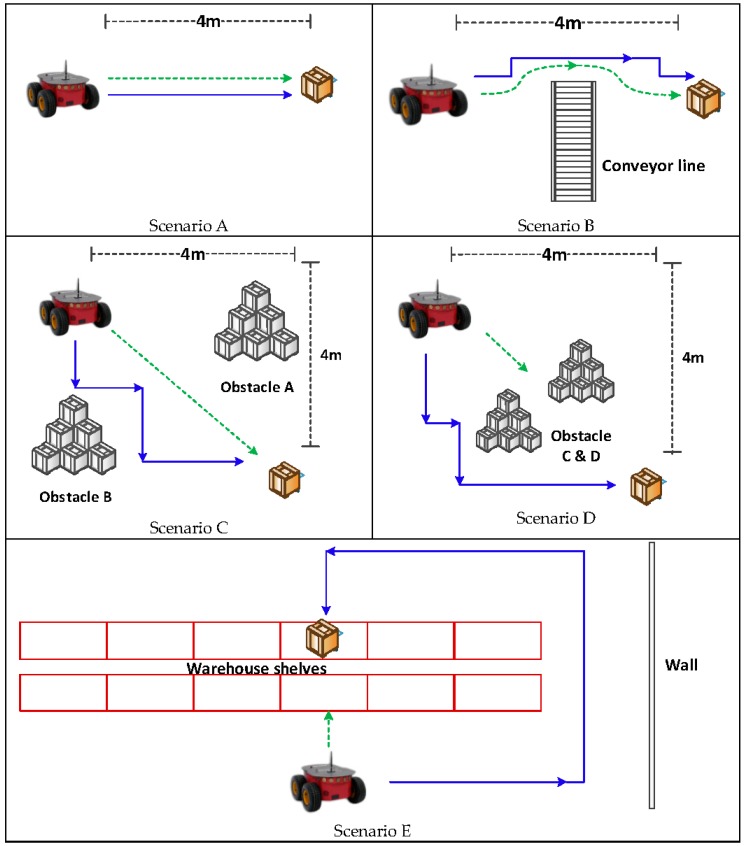
Diagrams showing the five test scenarios, with the expected paths of the preliminary system (green, dashed arrow) and the full system (blue, solid arrow).

Based on the motivating scenario, a number of benchmark tests were developed in order to gauge the performance of the system. Each test was designed to determine if the robot could complete a fundamental task such as driving straight and navigating around obstacles, and how quickly these tasks could be completed. These fundamental tasks indicate the ability to perform more complex operations in our motivating scenario, which is made up of different combinations of these fundamental tasks. These tests were performed using both the preliminary (with only target localisation and motion planning) and full (with mapping and path planning also) navigation systems, in order to determine if the final system outperforms the preliminary system. These tests varied in complexity to test a range of scenarios. For each test, the robot was considered to have completed the task when the robot was within 50 cm of the emitter (since the robot is 40 cm in diameter). These tests are presented diagrammatically in Figure 13.

Scenario A involves a straight-line test with no obstacles as a control—it was expected that both systems would perform identically since the robot should just drive in a straight line to the goal. Scenario B is a similar test but with an obstacle in the way, preventing a direct straight-line path. In this test, it was expected that the full navigation system would outperform the preliminary system as the full navigation system should be able to find a path around the obstacle much more easily with the A* path planning algorithm, whereas the preliminary system would need to rotate away from the obstacle and iteratively try to find its way around the obstacle. In both scenarios, the robot drives in a direction with increasing RSS values.

In Scenario C and D, the emitter was offset from the robot by 4 m in the x and y directions so that the emitter was at a 45° angle to the robot. Without any obstacles, it would be expected that the preliminary system would be able to drive in a straight-line to the goal, whereas the full system had to conform to the grid structure and would therefore drive along the perimeter of the square so that it makes a minimal number of turns. In Scenario C, we placed obstacles in the opposite corners of the area, so that this ideal L-shaped path is not possible. It was hypothesised that the preliminary system would be able to drive between the obstacles and arrive at the goal, whereas the full system would have to create a more complicated path. Due to there being a cost associated with turning, it was expected that the robot would not attempt to approximate a straight line, as this would require many turns and would not decrease the path sufficiently to balance out the increased turning cost. Similar to scenario A and B, the robot could reach the final target by simply driving in a direction with increasing RSS values. 

In Scenario D, the obstacles were placed so that they completely block the straight-line path to the emitter. It was expected that the full navigation system would outperform significantly in this test as the direct path to the goal is blocked, and the robot may need to drive away from the goal in order to avoid the obstacle. As the preliminary system has no global map or path, it has no ability to plot a path in what appears to be the opposite direction to the target in order to make overall progress. Due to this, it was expected to perform poorly and could be unable to complete the task.

Lastly, in Scenario E, we created a U-turn test that required the robot to drive in the opposite direction to the radio signal in order to avoid a large obstacle. It was expected that the preliminary system would face significant difficulties and may not even be able to reach the goal, as it had no memory of the positions of previously encountered obstacles. However, the full system should be able to find a path by exploring new unoccupied areas until the robot reached the end of the warehouse shelves and found a valid path to the goal. In both scenario D and E, the robot was required to move in a direction that is likely to have a lower RSS value initially, but the robot should eventually reach the target location through long-term path planning.

### 7.2. Results and Discussions

Each benchmark test was performed three times to ensure repeatability of the experiments and averaged, as shown in Table 1. In each experiment, the range of the results was negligibly small. In general, the hypothesis could be formulated that the preliminary system performs better in scenarios where there is a straight line to the emitter, and the full system performs better in all other cases. For four of the five tests, results were as expected. The unexpected result was that the preliminary system outperformed the full system in the blocked 4 m straight-line test (scenario B). This result may be due to small fluctuations in the estimate of the target location causing variations in the predicted path in the full system. In the blocked straight-line test, these small changes in the target position estimate caused the path-planning module to switch the path to go around the other side of the obstacle. If this fluctuation happens repeatedly, the robot may drive back and forth behind the obstacle without making any forward progress.

Overall, these results support the claim that the full system is able to navigate relatively complex environments, as it significantly outperforms the preliminary system in the two final tests. In particular, for the fully blocked 4 m right angle test (scenario D), the robot could only complete it successfully one time (i.e., 391 s for that single test rather than an average) when using the preliminary system, and the preliminary system could not complete the U-turn test at all. Without long-term path planning, the robot was not able to move away from the target emitter to find an alternative route that eventually led back to the emitter.

In a separate test, we tried to determine the maximum range of our transmitter/receiver in ideal conditions, and to determine if the system was still effective over long distances. This involved placing the transmitter at the end of a long corridor, with a transmission power of 0 dBm. The transmitter was able to be detected at a range of over 50 m, and the system was able to successfully navigate to the target over a period of approximately 20 min. However, this maximum distance could still be increased by using a higher transmission power.

## 8. Conclusions and Future Work

In this paper, we presented two main contributions: a novel algorithm for localising a single emitter using Received Signal Strength (RSS) from multiple directional antennae to derive direction estimates that improve upon existing localisation methods based only on distance estimates, and a system integration that demonstrates the effectiveness of this approach on a mobile robot. We developed a low-cost omnidirectional antennae setup with cylindrical parabolic reflector dishes that enabled the calculation of the direction of a signal source based on the angle of arrival. The received RSS values were processed with a particle filter in order to enable accurate target localisation, reducing the effects of interference and variations in RSS. This was combined with a simple motion planning system that allowed for basic obstacle avoidance while still progressing towards the end goal in some cases. This preliminary system was able to localise and navigate to the wireless emitter in open spaces and environments where there are minimal obstacles.

With the addition of a Simultaneous Localisation and Mapping (SLAM) module, a map of the environment was generated that enabled long-term path planning using the A* algorithm. This enabled the robot to perform autonomous navigation in much more complex environments with multiple obstacles, including scenarios where the robot has to drive away from the target position in order to move around obstacles. The improved performance is validated through a number of experimental tests designed according to factory automation scenarios that demonstrate the superiority of the full system when there is no straight-line path available.

The techniques described in this paper are applicable to a wide range of scenarios beyond industrial automation, such as scenarios with frequent changes and those that require the use of ad-hoc robotic systems. In emergency situations where successful deployment is time-critical, approaches that reduce the setup costs and times such as ours will be preferable to those that require significant infrastructure. Future work includes implementing more advanced forms of path planning to reduce the stringency of the current movement grid constraints to allow for more optimal navigation, improving the choice of obstacle avoidance sensors to allow for range finding in multiple planes, and potentially adapting the approach to allow for three-dimensional navigation by drones or underwater vehicles. Multipath effects could also be better estimated by identifying the shape and material of nearby obstacles, allowing for better correction of these effects at the receiver. This could also be achieved by incorporating other sensor data into the existing particle filter.

## Figures and Tables

**Table 1:** Benchmark results, with the best result in each test bolded.

Scenario	Mean Time (Sec) Taken in Preliminary System	Mean Time (Sec) Taken in Full System
(A) 4 m straight line	89	**90**
(B) Blocked 4 m straight line	126	230
(C) Partially blocked 4 m right angle	252	**225**
(D) Blocked 4 m right angle	391	**222**
(E) U-turn	Could not complete	**455**
